# Treatment strategies, radiological recovery, and neurodevelopmental outcomes in paediatric Maple Syrup Urine Disease: a 20-year single-centre experience from Türkiye

**DOI:** 10.1007/s11011-026-01896-8

**Published:** 2026-06-22

**Authors:** Kemal Uylaş, Havva Yazıcı, Yasemin Atik Altınok, Merve Yoldaş Çelik, Fehime Erdem Karapınar, Pınar Yazıcı Özkaya, Esra Isik, Cenk Eraslan, Ebru Canda, Ozgur Cogulu, Bülent Karapınar, Sara Habif, Mahmut Çoker, Sema Kalkan Uçar

**Affiliations:** 1 Department of Pediatrics, Ege University Medical Faculty, Bornova, Izmir, Türkiye; 2 Department of Radiology, Ege University Medical Faculty, Bornova, Izmir Türkiye; 3 Department of Biochemistry, Ege University Medical Faculty, Bornova, Izmir Türkiye

**Keywords:** Maple syrup urine disease, Sodium phenylbutyrate, Haemodialysis, Neurodevelopment, MRI, Branched-chain amino acids

## Abstract

Maple Syrup Urine Disease (MSUD) is a rare autosomal recessive metabolic disorder characterised by defective branched-chain amino acid (BCAA) catabolism, leading to neurotoxicity, recurrent metabolic crises, and neurodevelopmental impairment. Evidence on long-term outcomes in paediatric cohorts, particularly with pharmacological adjuncts such as sodium phenylbutyrate (NaPBA) and radiological recovery, remains limited. We undertook a retrospective review of 13 paediatric patients with MSUD (69.2% classic phenotype, 30.8% intermittent) followed at a tertiary metabolic centre in Türkiye between 2003 and 2022. Demographic, biochemical, neurodevelopmental, neuroimaging, and genetic data were evaluated, with specific attention to dietary management, haemodialysis during acute decompensation, and NaPBA therapy. All patients exhibited neurodevelopmental delay, which was more pronounced in the classic phenotype. Milestone-level analysis demonstrated delays in walking (85%), sentence formation (92.3%), and toilet training (92.3%). One year after dietary intervention, mean plasma concentrations of leucine, isoleucine, and valine decreased by 60.9%, 55.9%, and 65.0%, respectively (*p* < 0.01). Haemodialysis during metabolic crises rapidly reduced leucine (− 73.8%) and ammonia (− 66%), though was more frequently required in patients with the classic phenotype. NaPBA treatment was associated with lower leucine levels during follow-up (*p* < 0.05). Baseline MRI abnormalities were identified in 87% of patients; 57% showed complete resolution post-treatment, with partial radiological improvement observed alongside clinical follow-up. A phenotype-specific approach combining early dietary intervention, timely haemodialysis in acute crises, and selective use of NaPBA may support metabolic stabilisation and radiological improvement in selected patients. Larger multicentre studies are warranted to validate these findings and refine management protocols.

## Introduction

**Fig. 1:**
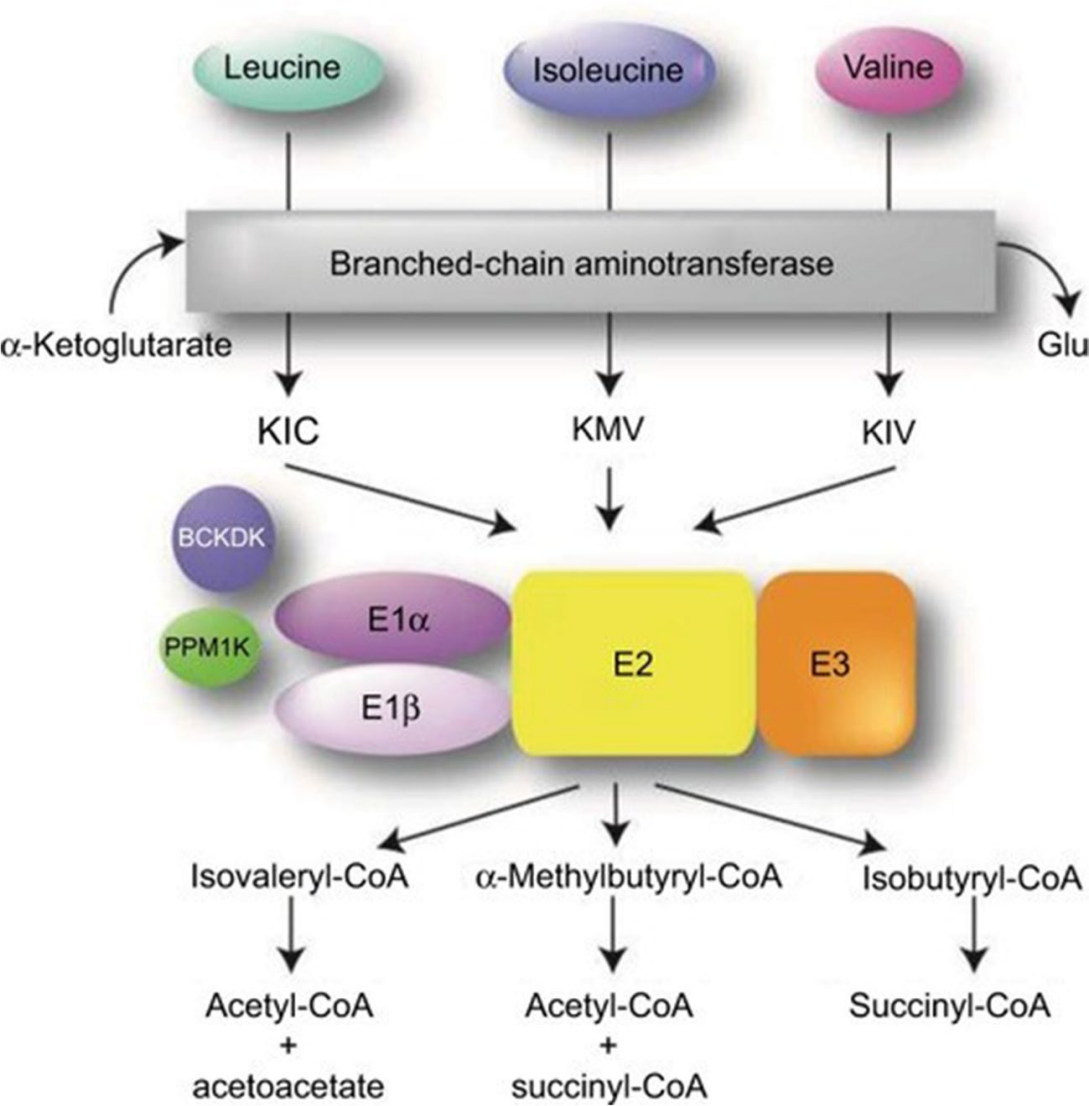
Overview of the BCAA catabolic pathway. Branched-chain amino acids undergo transamination, catalysed by branched-chain aminotransferase (BCAT) in the presence of α-ketoglutarate, producing the corresponding α-ketoacids—KIC, KMV, and KIV. These intermediates are subsequently oxidatively decarboxylated by the BCKAD complex. Abbreviations: KIC, α-ketoisocaproic acid; KIV, α-ketoisovaleric acid; KMV, α-keto-β-methylvaleric acid; Glu, glutamate; BCAAs, branched-chain amino acids; BCKAD, branched-chain α-ketoacid dehydrogenase (Blackburn et al. 2017)

Maple Syrup Urine Disease (MSUD; OMIM #248600) is a rare autosomal recessive inborn error of metabolism caused by deficiency of the branched-chain α-ketoacid dehydrogenase complex (BCKDC), a mitochondrial enzyme that catalyses the oxidative decarboxylation of the branched-chain amino acids (BCAAs) leucine, isoleucine, and valine (Fig. 1) (Hassan and Gupta 2025). These branched-chain amino acids are primarily utilised for energy production or to stimulate protein synthesis, including muscle growth, through activation of the mTOR signalling pathway (Dimou et al. 2022). Inadequate BCKDC activity results in accumulation of these amino acids and their corresponding ketoacids to neurotoxic concentrations, particularly affecting the central nervous system, and leading to encephalopathy, progressive neurodevelopmental impairment, and, if untreated, death (Blackburn et al. 2017). The disorder was first described in 1954 by Menkes and colleagues, who identified a distinctive sweet odour in the urine of affected neonates presenting with severe encephalopathy (Chuang 1998). It is now classified into several phenotypic subtypes such as classic, intermediate, intermittent, thiamine-responsive, and E3-deficient which are based on residual enzymatic activity, genotype, and clinical severity (Strauss et al. 2020). The classic form, with < 2% residual activity, is the most severe, typically presenting within the first days of life with poor feeding, lethargy, vomiting, dystonia, and metabolic acidosis, rapidly progressing to cerebral oedema, coma, and death without treatment (Strauss et al. 1993).

The estimated global incidence of MSUD is approximately 1 in 185,000 live births, but higher rates occur in populations with high consanguinity or founder effects, including Old Order Mennonites, Ashkenazi Jews, and Portuguese Gypsies (Edelmann et al. 2001). In Türkiye, incidence is presumed to be higher than the global average owing to elevated consanguinity rates (Younes et al. 2025). Effective management of MSUD necessitates meeting the patient’s nutritional requirements while ensuring optimal control of acute metabolic decompensations (Hallam et al. 2005). Treatment involves a protein-restricted diet supplemented with synthetic, BCAA-free amino acid formulas enriched with essential amino acids, vitamins, and micronutrients. This strategy aims to maintain plasma BCAA concentrations within target ranges to prevent metabolic decompensation while supporting normal growth and development (Frazier et al. 2014). Acute metabolic decompensation, typically at plasma leucine levels > 380 mmol/L, are often triggered by dietary non-compliance or infection (Tu et al. 2025). Decompensation arises more commonly in the first year of life and after age 15 (Abi-Wardé et al. 2017). This event requires urgent intervention; high-caloric intake, intravenous fluids, insulin, and, in severe cases, extracorporeal detoxification via continuous renal replacement therapy (CRRT) techniques such as haemodialysis or haemofiltration (Hassan and Gupta 2025).

Adjunct pharmacological strategies have recently emerged. Sodium phenylbutyrate (NaPBA), developed for urea cycle disorders, has been shown to promote dephosphorylation and activation of BCKDC, potentially enhancing residual enzymatic activity (Burrage et al. 2014). While not yet standard of care, early clinical observations suggest NaPBA may contribute to improved metabolic control (Zubarioglu et al. 2021). Liver transplantation offers a definitive source of functional BCKDC activity and can normalise metabolic control, abolishing dietary restrictions and preventing future crises; however, it does not reverse pre-existing neurological injury and carries risks of surgery and lifelong immunosuppression (Aras et al. 2023). Neurodevelopmental outcomes are strongly influenced by the timing of diagnosis, the severity and frequency of crises, and the consistency of metabolic control. Even with newborn screening and optimal management, many patients, particularly those with the classic phenotype, experience residual developmental delays, behavioural challenges, and reduced quality of life (Magdy et al. 2025). This study aims to describe the clinical features, treatment strategies, and outcomes of 13 paediatric MSUD patients followed at a tertiary metabolic centre over two decades, with particular focus on dietary therapy, NaPBA use, and haemodialysis. These findings are discussed in the context of contemporary international evidence to inform both national practice and global MSUD management strategies.

## Methods

### Study design, setting, and participants

This retrospective observational study was conducted at the Paediatric Metabolism Division of Ege University Faculty of Medicine, Department of Paediatrics, between 2003 and 2022. The study included 13 patients diagnosed with Maple Syrup Urine Disease (MSUD), diagnosed by clinical presentation, elevated branched-chain amino acids, and confirmed by molecular genetic analysis. All patients were under follow-up at the same centre during the study period. Data were obtained from patient case files archived in the metabolic clinic and hospital laboratory records. For each patient, a standardised Case Report Form (CRF) was completed and verified by the principal investigator or an authorised member of the research team. Clinical, radiological and laboratory data were analysed and compared both within the cohort and with findings from the literature. The study protocol was approved by the Ege University Medical Research Ethics Committee (approval date: 09.09.2022, decision number: 22-9 T/30). Written informed consent for use of anonymised clinical data was obtained from parents or guardians.

### Classification of MSUD

MSUD can be classified into classic (cMSUD; ≤ 2% residual enzymatic BCKDH activity) and variant forms (vMSUD; 3%−40% residual enzymatic BCKDH activity). cMSUD accounts for the majority of cases and leads to a more severe and less variable clinical phenotype than vMSUD. Biochemically, the diagnosis of MSUD is established by detection of elevated concentrations of BCAA (with leading hyperleucinosis) and allo-isoleucine in plasma, and their corresponding BCKA in urine. The determination of enzymatic activity in enzyme assays can be used to further distinguish between cMSUD and vMSUD. Phenotypic classification in this cohort was based on age at onset, biochemical severity, and clinical course.

### Laboratory and diagnostic analyses

As part of the laboratory evaluation, plasma amino acids were measured by LC–MS/MS (SCIEX Triple Quad 3500, AB Sciex) after protein precipitation and centrifugation, with quantification by internal standard calibration (intra-assay CV < 12%, inter-assay CV < 15%). Plasma ammonia was analyzed enzymatically on a Cobas 8000 system (Roche Diagnostics) using a GLDH-based kinetic method. Normal ranges: leucine 75–175 µmol/L, isoleucine 25–75 µmol/L, valine 150–300 µmol/L.

MRI scans were performed using a standard clinical protocol on a 1.5 Tesla system. Sequences included T1-weighted, T2-weighted, and FLAIR images in axial, coronal, and sagittal planes. All scans were independently evaluated by an experienced neuroradiologist, and findings were interpreted in the context of the clinical presentation.

Finally, to determine the molecular etiology, all four known causative genes of Maple Syrup Urine Disease (*BCKDHA, BCKDHB, DBT, and DLD*) were systematically analyzed and variants were classified according to ACMG guidelines. Genetic testing was performed using a targeted next-generation sequencing (NGS) panel encompassing the complete coding regions and exon–intron boundaries of these genes. Sequence data were aligned to the human reference genome (GRCh37/hg19), and variants were called, annotated, and interpreted in accordance with the American College of Medical Genetics and Genomics (ACMG) guidelines. Putative pathogenic variants were confirmed by Sanger sequencing.

### Acute-crisis management

The acute management of metabolic crises in maple syrup urine disease (MSUD) aims to rapidly reduce elevated branched-chain amino acid (BCAA) and branched-chain ketoacid levels, prevent cerebral edema, and restore metabolic stability. The initial steps involve immediate cessation of natural protein intake, reversal of catabolism through high-caloric glucose and lipid infusions, and provision of BCAA-free amino acid formulas to support anabolism (Hassan and Gupta 2025). In severe crises with high leucine levels (> 1500–2000 µmol/L), extracorporeal detoxification methods such as haemodialysis or haemodiafiltration are indicated due to their superior efficiency in rapidly lowering leucine concentrations compared with peritoneal dialysis (Strauss et al. 2020).

### Exclusion criteria

Patients were excluded if they had incomplete data due to irregular follow-up, discontinued follow-up at the study centre or no molecular confirmation of the MSUD diagnosis.

### Data collection procedures

Data were extracted from outpatient records and hospital laboratory systems. The following variables were recorded:

- Demographic characteristics (age, sex, birth details).
- Anthropometric measurements (weight, height, head circumference).
- Quantitative plasma amino acid levels (leucine, isoleucine, valine).
- Plasma ammonia levels.
- Recommended and actual intake of BCAAs (leucine, isoleucine, valine).
- Daily protein and caloric intake.

Nutritional data were taken only from outpatient follow-up visits; dietary plans implemented during inpatient admissions were excluded. Anthropometric data were evaluated using the Turkish growth charts developed by Neyzi et al., accessed via the ÇEDD-ÇÖZÜM online platform of the Turkish Paediatric Endocrinology and Diabetes Society. Children’s neuromotor development was systematically assessed during scheduled follow-up visits, generally conducted at intervals of several months. Developmental milestones were evaluated using parental reports and direct clinical observation during physical examinations. This combined approach enabled consistent longitudinal monitoring of milestone acquisition over time.

### Statistical analysis

Data were analysed using SPSS software version 25.0 (IBM Corp., Armonk, NY). Descriptive statistics were expressed as frequency, percentage, mean, median, standard deviation, minimum, and maximum values. The Shapiro–Wilk test was used to assess normality. For comparisons between two independent groups with non-normally distributed data, the Mann–Whitney U test was applied. For comparisons involving more than two independent groups, the Kruskal–Wallis H test was used. The Wilcoxon signed-rank test was used for paired non-parametric data. Correlations between continuous variables were analysed using the Spearman correlation coefficient (*r*). A *p*-value less than 0.05 was considered statistically significant for all analyses, with a 95% confidence interval. No formal power calculation was performed prior to data collection. Due to the small cohort size (*n* = 13), p-values should be interpreted descriptively rather than confirmatorily, and inter-group comparisons (e.g., classic versus intermittent phenotype) should be considered exploratory and likely underpowered.

## Results

### Patient demographics & presentation

Thirteen patients were included in the study, comprising nine (69.2%) with the classic phenotype and four (30.8%) with the intermittent phenotype. Seven patients (53.8%) were female and six (46.2%) male. The median age at diagnosis was 12.5 ± 20 months, ranging from 1 to 60 months. The mean age of diagnosis was 4.8 ± 3.6 months in classical subtype cases and 30 ± 31.3 months in intermittent type cases. The mean age of the surviving patients was 10 years (Table 1). Consanguinity was reported in 69.2% (9/13) families. Birth weights were within the normal range for gestational age in all patients.

**Table 1:** Distribution of MSUD cases according to phenotype

Phenotype	*N*	%	Mean ± SD (months)	I.Q–3.Q (months)	Median (months)	Min–Max (months)
Classic	9	69.2	4.8 ± 3.6	1.5–8.5	3	1–9
Intermittent	4	30.8	30 ± 31.3	2–58.5	29.5	1–60
Total	13	100	12.5 ± 20	1.5–9	5	1–60

The most common presenting symptom was poor feeding (53.8%), followed by hypertonia (46.1%) and encephalopathy (38.4%). When evaluating symptoms across MSUD phenotypes, the distribution of presenting features varied between classic and intermittent subtypes. Encephalopathy and spasticity occurred only in classic MSUD while hypertonia was more common in intermittent than classic (50.0% vs 44.4%). While differences in symptom distribution such as the presence of encephalopathy and spasticity only in the classic phenotype were clinically notable, they did not reach statistical significance due to the small sample size (Table 2).

**Table 2:** Presenting symptoms by phenotype

Symptom	Total *N*	Classic *N*	Classic %	Intermittent *N*	Intermittent %
Feeding difficulty	7	6	66.7	1	25.0
Hypertonia	6	4	44.4	2	50.0
Encephalopathy	5	5	55.6	0	0.0
Vomiting	4	3	33.3	1	25.0
Hypotonia	3	3	33.3	1	25.0
Spasticity	2	2	22.2	0	0.0
Metabolic acidosis	1	1	11.1	0	0.0

At presentation, all patients exhibited biochemical profiles typical of MSUD, including elevated plasma levels of leucine, isoleucine, valine, and allo-isoleucine, as well as the presence of ketonuria and mild hyperammonemia in some cases.

### Neurodevelopmental & growth outcomes

Neuromotor development was assessed across nine milestones: smiling, eye tracking, head control, crawling, sitting without support, walking, single-word speech, sentence construction, and toilet training. At baseline, delays were common, affecting ≥ 60% of patients for most milestones. Walking (84.6%, *n* = 11), sentence construction (92.3%, *n* = 12), and toilet training (92.3%, *n* = 12) showed the highest rates of delay. In contrast, smiling, eye tracking, and head control were delayed in only 38.4% (*n* = 5) of patients. All patients eventually achieved smiling, eye tracking, and head control, with median acquisition ages of 3 months (range 1–28), 3 months (1–28), and 5 months (1–28), respectively. For motor milestones, unsupported sitting was achieved by all 13 patients, but with a median age of 12 months (5–72) and delay in 76.9% (*n* = 10). Walking was achieved by 54% (*n* = 7), with a median age of 20 months (10–72). Speech delays were notable: only 7.7% (*n* = 1) spoke a single word or formed sentences on time, and 7.7% (*n* = 1) achieved timely toilet training. By follow-up, 92.3% (*n* = 12) had acquired single-word speech (median 20 months, 10–72), 53.8% (*n* = 7) sentence construction (median 36 months, 24–60), and 61.5% (*n* = 8) toilet training (median 48 months, 24–120). These findings demonstrate substantial developmental delay in the cohort, particularly in gross motor functions and language acquisition. Delays were not only frequent but also protracted in terms of age of milestone acquisition.

Phenotype-specific analysis showed greater delays and later acquisition in the classic group compared with the intermittent group for most milestones. For example, crawling was achieved in 66.7% (*n* = 6) of classic patients at a mean of 31.5 ± 23.9 months versus 100% (*n* = 4) of intermittent patients at 9.0 ± 2.0 months; walking in 44.4% (*n* = 4) of classic patients at 38.7 ± 28.9 months versus 100% (*n* = 4) of intermittent patients at 18.0 ± 8.5 months; and sentence formation in 33.3% (*n* = 3) of classic patients at 42.0 ± 15.9 months versus 100% (*n* = 4) of intermittent patients at 42.0 ± 15.5 months. These findings suggest that developmental delays were more pronounced in classic MSUD, particularly in gross motor and expressive language milestones.

#### Weight

Treatment significantly improved weight-for-age SDS in the first year (*p* = 0.032).

#### Height

A significant decline in height-for-age SDS was observed by the fourth year of treatment (*p* = 0.005), indicating a long-term impact on linear growth.

#### Head circumference

While not statistically significant, classic-type patients had smaller head circumference SDS, correlating with more severe neurological involvement.

### Biochemical & therapeutic efficacy

#### Long-term metabolic control (Diet)

Excluding measurements taken during acute metabolic crises, mean plasma branched-chain amino acid (BCAA) levels were monitored at baseline, 6 months, 1 year, 2 years, 4 years, and 8 years following the initiation of dietary treatment. For leucine, mean concentrations were 323 μmol/L at baseline, 301 μmol/L at 6 months, 324 μmol/L at 1 year, 332 μmol/L at 2 years, 334 μmol/L at 4 years, and 334 μmol/L at 8 years. No statistically significant changes were observed when compared either with baseline or sequentially year-on-year (all *p* > 0.05). For isoleucine, corresponding values were 156, 135, 139, 145, 136, and 145 μmol/L. Differences from baseline were not statistically significant (all *p* > 0.05), although a significant increase was detected at year 4 compared with the previous year (*p* = 0.033). For valine, mean values were 154, 143, 158, 181, 193, and 190 μmol/L. No significant changes from baseline were observed at 6 months, 1 year, or 2 years (all *p* > 0.05). However, levels at year 4 (*p* = 0.044) and year 8 (*p* = 0.042) were significantly higher than baseline. Additionally, valine increased significantly from year 1 to year 2 (*p* = 0.039) and from year 2 to year 4 (*p* ≤ 0.025). These findings suggest that long-term dietary therapy maintained relatively stable leucine and isoleucine levels outside acute episodes, whereas valine levels showed a gradual upward trend over extended follow-up.

In our cohort, when cases were stratified into classical and intermittent subtypes, long-term follow-up measurements (excluding periods of metabolic crisis) of leucine, isoleucine, valine, and ammonia were assessed. No significant differences were observed between the classical and intermittent subtypes in leucine, isoleucine, or valine levels during follow-up (*p* > 0.05). However, ammonia levels showed a significant difference within the intermittent subtype (*p* = 0.031). Detailed values for leucine, isoleucine, valine, and ammonia in the long-term follow-up of both subtypes are presented in Table 3.

**Table 3:** Leucine, isoleucine, valine, and ammonia levels in classical and intermittent subtype patients during long-term follow-up excluding attack periods

	Classical		Intermittent		*p*-value
	Mean ± SD	Median	Mean ± SD	Median	
Leucine (μmol/L)	367.0 ± 150.4	296.0	218.8 ± 76.0	246.5	0.123
Isoleucine (μmol/L)	160.1 ± 49.7	154.0	114.3 ± 47.0	125.0	0.217
Valine (μmol/L)	181.7 ± 64.6	166.0	229.0 ± 54.4	232.5	0.217
Ammonia (μg/dL)	77.3 ± 19.3	73.0	53.0 ± 7.9	53.5	**0.031**

#### Acute decompensation management

During metabolic crises, 13 patients underwent either an acute BCAA-depletion (elimination) diet alone (*n* = 8) or in combination with haemodialysis (*n* = 5). Patients requiring haemodialysis (all classic subtype) represented a more severe cohort, with a significantly higher frequency of metabolic attacks compared to those not requiring haemodialysis (*p* = 0.038). This group also presented with a lower mean head circumference SDS (−2.20 vs. −0.60) and a higher median number of therapeutic washout diets (7 vs. 3), although these differences were not statistically significant.

##### Acute BCAA-depletion (Elimination Diet)

This intervention was associated with marked reductions in plasma BCAA concentrations. Across all patients, the mean leucine level decreased from 1141 µmol/L at baseline to 445 µmol/L by the third day (*p* = 0.0001). Similarly, significant reductions were observed for isoleucine (386 µmol/L to 170 µmol/L; *p* = 0.0001) and valine (607 µmol/L to 212 µmol/L; *p* = 0.0001).

##### Haemodialysis

During the follow-up period, haemodialysis was performed in eight patients (61.5%) for the management of severe metabolic decompensation. All haemodialysis interventions were conducted during hospital admissions triggered by metabolic crises. This intervention was associated with a mean reduction of 54% in leucine (from 1394 to 635 µmol/L), 49% in isoleucine (from 513 to 258 µmol/L), 43% in valine (from 665 to 374 µmol/L), and 20% in ammonia (from 82 to 65 µg/dL) (all *p* = 0.043). Patients undergoing haemodialysis experienced a higher annual attack frequency compared with those treated with diet alone (0.88 vs 0.41 attacks/year; *p* = 0.038). No significant differences were found in anthropometric z-scores between the two groups. Intermittent subtype patients did not require haemodialysis.

#### Sodium phenylbutyrate (NaPBA) therapy

Seven patients received NaPBA during long-term follow-up outside of attack periods. Comparison of mean leucine, isoleucine, valine, and ammonia levels between NaPBA users and non-users showed no statistically significant differences (*p* > 0.05). Within the NaPBA-treated group, pre- and post-treatment values were as follows: leucine 321 μmol/L vs 305 μmol/L, isoleucine 146 μmol/L vs 123 μmol/L, valine 199 μmol/L vs 166 μmol/L, and ammonia 70 μg/dL vs 73 μg/dL (Table 4). A statistically significant reduction was detected only for leucine (*p* = 0.043), while changes in isoleucine, valine, and ammonia were not significant (*p* > 0.05).

**Table 4:** Mean leucine, isoleucine, valine, and ammonia values in patients using and not using NaPBA

	NaPBA users (Mean ± SD)	Median	NaPBA non-users (Mean ± SD)	Median	*p*
Leucine (μmol/L)	305 ± 147.0	230.0	304.9 ± 147.0 μmol/L	272.5	0.886
Isoleucine (μmol/L)	123.1 ± 51.4	103.0	123.1 ± 51.4 μmol/L	133.5	0.668
Valine (μmol/L)	166.4 ± 67.1	164.0	166.4 ± 67.1 μmol/L	206.0	0.199
Ammonia (μg/dL	73.0 ± 32.2	60.0	73.0 ± 32.2 μg/dL	63.0	0.774

When stratified by phenotype, annualised attack frequency remained unchanged pre- and post-NaPBA in both classical (0.5/year) and intermittent (0.2/year) subtypes. In both subtypes, no significant differences were observed following NaPBAin mean leucine, isoleucine, or valine concentrations (*p* > 0.05). However, ammonia levels decreased significantly following NaPBA across both subtypes (*p* = 0.034).

### Genetic & radiological findings

#### Genetic profile

Genetic analysis revealed that *BCKDHB* mutations were the most prevalent (10/13 patients; 76.9%), followed by *DBT* (15%) and *BCKDHA* (7.5%). Notably, four novel, previously unreported pathogenic variants were identified (Table 5), expanding the known mutational spectrum of MSUD. In the classical subgroup, 53% (seven) cases had a mutation in the *BCKDHB* gene, and 15% (two) cases had a mutation in the *DBT* gene. According to the data of four cases in the intermittent subgroup, it was observed that 7% (one) case had *BCKDHA* gene mutation and 23% (three) cases had *BCKDHB* gene mutation. No mutation in the *DLD* gene was detected in the cases included in the study. Table 5 shows the gene distribution of the cases. No mutation in the *DLD* gene was detected in the cases included in the study.

**Table 5:** Distribution of mutations, ACMG classifications, and clinical segregation in the study group

Patient	Gene	cDNA	Protein	Genotype/Zygosity	ACMG class	Applied criteria
1	*BCKDHB*	c.951 + 1G > T; c.93_103dupGGCCGCGGGGCT	p.?; p.Phe35fs	Compound Heterozygous	Likely Pathogenic	PVS1, PM2
2	*BCKDHB*	**c.509_510delGC**	**p.Arg170fs**	Homozygous	Likely Pathogenic	PVS1, PM2
3	*DBT*	c.1018-1G > A	p.?	Homozygous	Pathogenic	PVS1, PM2, PS3
4	*BCKDHB*	c.1016C > T	p.Ser339Leu	Homozygous	Pathogenic	PS1, PM2, PP3
5	*BCKDHB*	c.1006G > A	p.Gly336Ser	Homozygous	Likely Pathogenic	PM2, PP3, PP2
6	*BCKDHB*	c.547 C > T	p.Arg138Trp	Homozygous	Pathogenic	PS1, PM2, PP3
7	*DBT*	c.898 delG; c.1190C > T	p.Ala300LeufsX13; p.Thr397Ile	Compound Heterozygous	Likely Pathogenic	Allele 1: PVS1 Allele 2: PM2, PP3
8	*BCKDHB*	c.1149T > A; **c.400delA**	p.Tyr383X; **p.Ile134fs**	Compound Heterozygous	Likely Pathogenic	PVS1, PM2
9	*BCKDHB*	c.564T > A	p.Cys188X	Homozygous	Pathogenic	PVS1, PM2
10	*BCKDHB*	**c.722A > T**	**p.Lys241Ile**	Homozygous	VUS	PM2, PP3, PP2
11	*BCKDHB*	c.1016C > T; **c.1090G > A**	p.Ser339Leu; **p.Asp364Asn**	Compound Heterozygous	Likely Pathogenic	PM2, PM3, PM1, PP2, PP3
12	*BCKDHA*	c.890G > A	p.Arg297His	Homozygous	Pathogenic	PS1, PM2, PP3
13	*BCKDHB*	c.832G > A	p.Gly278Ser	Homozygous	Pathogenic	PS1, PM2, PP3

None of the four novel BCKDHB variants identified in our cohort had been previously reported in public archives, including ClinVar and HGMD. Based on the ACMG/AMP consensus guidelines, their pathogenicity was classified as follows. The novel homozygous c.509_510delGC (p.Arg170Leufs*6) variant was identified in patient 2 and classified as Likely Pathogenic based on PVS1 (loss-of-function) and PM2 (absence in gnomAD) criteria. While the genetic findings aligned with the clinical presentation of the patients classic MSUD phenotype, familial segregation supported pathogenicity; both parents were confirmed asymptomatic heterozygous carriers, and the family history revealed an older sibling who had succumbed to MSUD complications. In patient 8, a classic MSUD phenotype was linked to the novel c.400delA (p.Ile134Serfs*96) variant, detected in a compound heterozygous state with c.1149 T>A (p.Tyr383X). Based on the PVS1 (frameshift-induced premature stop codon) and PM2 (absence in gnomAD) criteria, it was classified as Likely Pathogenic. Autosomal recessive segregation was verified by molecular testing of both parents, who were asymptomatic heterozygous carriers. A categorization of Variant of Uncertain Significance (VUS) was assigned to the novel homozygous c.722A>T (p.Lys241Ile) missense variant found in patient 10. This classification relied on PM2 (absence in gnomAD), PP2 (missense-sensitive gene domain), and PP3 (damaging in silico predictions) criteria. Consistent with the patient's intermittent MSUD phenotype, familial segregation supported its clinical relevance, with both parents confirmed as asymptomatic heterozygous carriers. Compound heterozygosity for the novel c.1090G>A (p.Asp364Asn) missense variant and the known pathogenic c.1016C>T (p.Ser339Leu) variant defined the genetic profile of patient 11. Classified as Likely Pathogenic based on PM2 (absence in gnomAD), PM3 (detected in trans), PM1 (critical functional domain), PP2, and PP3 criteria, this genotype was consistent with the intermittent MSUD phenotype. Pathogenicity was further corroborated by confirming that both parents were asymptomatic and heterozygous carriers. Genetic mutations and changes in proteins of 13 patients participating in the study are divided into classical subtype and intermittent subtype and given in Table 5.

*ACMG* American College of Medical Genetics and Genomics, *VUS* variant of uncertain significance, *MSUD* maple syrup urine disease

#### Neuroimaging (Cranial MRI)

Cranial MRI was available pre-treatment for most patients, though some scans were missing due to follow-up abroad or lack of recent imaging. In the classical subtype (*n* = 7 with imaging), pre-treatment abnormalities typically involved the globus pallidus, thalamus, mesencephalon, posterior pons, medulla oblongata, cerebellum, and corticospinal tracts, often with additional involvement of the centrum semiovale, dentate nuclei, corpus callosum, and periventricular white matter. Post-treatment, three patients achieved complete radiological normalisation, one showed partial regression of basal ganglia and thalamic lesions, and one developed persistent abnormalities in the pons, posterior mesencephalon, fourth ventricle, and bilateral globus pallidus. In the intermittent subtype (*n* = 4 with pre-treatment imaging), lesions were localised to the medial thalamus, globus pallidus, mesencephalon, and posterior pons. Post-treatment MRI was unavailable in three cases; the single patient with comparative scans demonstrated complete resolution of bilateral cerebral hemispheric, globus pallidus, and putamen signal changes. Overall, these findings indicate a high frequency of characteristic intramyelinating oedema at presentation, with potential for complete or partial resolution following effective metabolic control.

## Discussion

This single-centre cohort describes the long-term clinical, biochemical, and neurodevelopmental outcomes of 13 patients with MSUD in Türkiye, providing insight into disease expression and management in a high-consanguinity population. Our data confirm the absence of gender bias in MSUD, with an even distribution between males and female patients. The high rate of consanguinity (69.2%) reflects the well-recognised role of autosomal recessive inheritance in populations with frequent intrafamilial marriage (Tabbouche et al. 2014). Our consanguinity rate of 69.2% is consistent with MENAT-wide estimates (~ 72.5%), supporting the link between high homozygosity and the predominance of rare, region-specific variants in MSUD (Younes et al. 2025). Classic MSUD predominated (69.2%), as expected given its more severe enzymatic deficiency (Mitsubuchi et al. 2005). Diagnosis occurred later than in newborn screening (NBS) settings; mean 4.8 months in the classic subtype versus ~ 9.5 days with NBS in other series, particularly for intermittent cases, underlining the importance of optimising NBS algorithms to detect milder phenotypes (Couce et al. 2015). Malnutrition, spasticity, and encephalopathy were the most common presenting features in our cohort, consistent with other series (Blackburn et al. 2017; Margutti et al. 2020). Spasticity and encephalopathy were confined to the classic subtype, reflecting earlier and more severe CNS involvement (Blackburn et al. 2017). All classic patients experienced delays in gross motor and language milestones, with some never acquiring walking or toilet training. In contrast, all intermittent patients ultimately attained key milestones. These findings support published data that early detection and adherence to therapy improve outcomes, but severe phenotypes still carry a substantial risk of neurodevelopmental impairment (Scharre et al. 2025). Wider mechanistic research further highlights that neurological vulnerability in paediatric patients may reflect the convergence of multiple pathways, including disruption of axonal guidance signalling and cortical development (Yang et al. 2024; Qi and Guan 2024), and exposure to environmental neurotoxicants with neurodevelopmental consequences (Sun et al. 2025), reinforcing the importance of vigilant long-term neurodevelopmental surveillance in this population.

Body weight Z-score improved significantly in the first year after diagnosis, suggesting effective nutritional rehabilitation, a finding echoed in other intoxication-type metabolic disorders. Height Z-score declined during long-term follow-up, with partial recovery in adolescence, while head circumference tended to be smaller in classic cases. Growth impairment has been widely reported in MSUD and other inherited metabolic disorders requiring dietary restriction (Anton-Păduraru et al. 2025; Busiah et al. 2024).

Dietary therapy maintained relatively stable metabolic control in plasma leucine (− 60.9%), isoleucine (− 55.9%), and valine (− 65.0%), with most patients reaching guideline targets (Frazier et al. 2014). The lowest mean leucine concentrations were observed within the first six months, suggesting that adherence declines over time due to palatability, variety, and cost barriers; factors previously reported to limit long-term compliance (Avila and Abacan 2024). Acute decompensation was frequent in classic MSUD. Acute BCAA-depletion diets (elimination diet) were associated with reductions in BCAA levels, while haemodialysis (used exclusively in severe classic cases) achieved rapid leucine clearance (771–2041 µmol/L pre-haemodialysis to markedly lower post-haemodialysis), with concurrent ammonia reduction. Our findings were consistent with prior reports describing rapid biochemical improvement of extracorporeal detoxification in metabolic crises (Avila and Abacan 2024; Chanchlani et al. 2025). In a 2019 cohort study of neonates with inborn errors of metabolism, including five cases of MSUD among 40 participants, continuous venovenous haemodiafiltration (CVVHDF) was more effective than peritoneal dialysis in removing toxic metabolites; however, this advantage did not translate into improved survival outcomes (Celik et al. 2019). The haemodialysis group experienced more frequent crises, reflecting underlying disease severity. NaPBA was used in seven patients (six classic, one intermittent), producing a statistically significant long-term reduction in leucine concentrations without notable changes in other metabolites. Our NaPBA experience aligned with a Turkish single-centre series by Zubarioglu et al., who managed 19 acute MSUD episodes in 10 patients exclusively with medical therapy (Zubarioglu et al. 2021). Despite initial leucine levels often approaching CRRT thresholds, mean reductions of ~ 529 µmol/L/day at 24 h and ~ 319 µmol/L/day at 48 h were achieved, without extracorporeal clearance. While these rates are lower than those reported for CVVHDF, they are clinically meaningful and suggest a potential role for NaPBA as an adjunct in settings where CRRT is not feasible. These findings are consistent with emerging evidence that NaPBA can modulate BCAA metabolism, possibly via activation of the BCKDC complex, but its impact on clinical outcomes remains uncertain (Burrage et al. 2014; Brunetti-Pierri et al. 2011). Further controlled trials are warranted before widespread adoption.

*BCKDHB* mutations predominated (76.9%), followed by DBT (15%) and *BCKDHA* (7%). Several variants were reported for the first time, adding to the global mutation spectrum (Younes et al. 2025) and reinforcing the value of local genotyping for epidemiology and genetic counselling. Our identification of novel *BCKDHB* and *DBT* variants parallels recent MENAT-wide data showing that over three-quarters of MSUD-associated variants in the region are unique, with Türkiye contributing several population-specific alleles (Younes et al. 2025). This underscores the value of regional variant curation for targeted screening. MRI abnormalities were present in 87% at baseline, primarily affecting deep grey matter and brainstem structures, consistent with the known radiologic signature of MSUD (Li et al. 2021). Post-treatment imaging normalised in over half of cases, with partial regression or persistence in others. These findings are consistent with partial reversibility of MSUD-associated white matter lesions with timely metabolic control, though incomplete recovery underscores the vulnerability of the developing brain (Blackburn et al. 2017).

Key strengths of this study include its extended follow-up period, detailed phenotypic and biochemical profiling, and the use of multiple complementary modalities, clinical milestones, biochemical indices and neuroimaging to evaluate outcomes. Limitations include the small cohort size (*n* = 13), which is inherent to rare disease research; no formal power calculation was performed prior to data collection, and inter-group comparisons between the classic and intermittent subtypes are likely underpowered. The retrospective, single-centre design may limit the generalisability of findings. No formal adjustment for multiple statistical comparisons was performed; therefore, statistically significant findings should be interpreted cautiously and considered exploratory rather than confirmatory. Neurodevelopmental outcomes were assessed retrospectively using clinical follow-up documentation and milestone acquisition rather than standardised neurocognitive assessment tools, which may limit sensitivity for subtle impairment and introduce inter-observer variability. In addition, although ACMG classification criteria and familial segregation analyses supported pathogenicity of the novel variants identified, no functional enzyme assays or in vitro validation studies were performed. Moreover, NaPBA administration was not randomised; therefore, the observed biochemical benefits should be interpreted cautiously until confirmed by prospective, controlled studies. Overall, these results reinforce the value of a multidisciplinary management strategy for paediatric MSUD, encompassing early diagnosis, individualised dietary intervention, timely extracorporeal detoxification during metabolic crises, and judicious use of pharmacological adjuncts such as NaPBA in appropriately selected patients. Future priorities include conducting controlled trials of NaPBA in children, establishing multicentre registries to validate MRI recovery as a prognostic marker, and developing standardised neurodevelopmental assessment frameworks for long-term follow-up.

## Conclusions

This single-centre cohort from Türkiye provides a comprehensive overview of the clinical, biochemical, neurodevelopmental, and genetic features of paediatric MSUD in a high-consanguinity population. Our findings highlight the predominance of the classic phenotype, frequent acute metabolic decompensations, and the persistent neurodevelopmental challenges despite ongoing therapy. Novel pathogenic variants were identified, reinforcing the value of local genotyping for epidemiology and targeted screening. Nutritional management was associated with improved weight trajectories, but long-term growth and neurodevelopmental outcomes remain suboptimal. NaPBA was associated with biochemical changes that warrant further investigation and, in line with national experience, may represent a valuable adjunct in acute crises where extracorporeal detoxification is unavailable. No mortality was observed during follow-up in this cohort. Priorities for future work include expansion of universal newborn screening, optimisation of long-term dietary adherence, multicentre evaluation of NaPBA efficacy, and validation of MRI recovery as a prognostic tool.

## Data Availability

The data that support the findings of this study are not publicly available due to ethical and privacy restrictions related to patient confidentiality. De-identified data are available from the corresponding author upon reasonable request and subject to approval by the relevant institutional ethics committee.

## Abbreviations

**BCAA**: Branched-chain amino acids
**BCAT**: Branched-chain aminotransferase
**BCKA**: Branched-chain α-ketoacid
**BCKAD**: Branched-chain α-ketoacid dehydrogenase complex
**BCKDC**: Branched-chain α-ketoacid dehydrogenase complex (alternate abbreviation)
**BDK**: Branched-chain α-ketoacid dehydrogenase kinase
**CNS**: Central nervous system
**CRRT**: Continuous renal replacement therapy
**CVVH**: Continuous venovenous haemofiltration
**CVVHD**: Continuous venovenous haemodialysis
**CVVHDF**: Continuous venovenous haemodiafiltration
**DNPH**: Dinitrophenylhydrazine
**ILE**: Isoleucine
**KIC**: α-Ketoisocaproate
**KIV**: α-Ketoisovalerate
**KMV**: α-Keto-β-methylvalerate
**LEU**: Leucine
**MENAT**: Middle East, North Africa and Türkiye
**MRI**: Magnetic resonance imaging
**MSUD**: Maple syrup urine disease
**NaPBA**: Sodium phenylbutyrate
**NBS**: Newborn screening
**SDS**: Standard deviation score
**VAL**: Valine
